# Biogeography of dinoflagellate cysts in northwest Atlantic estuaries

**DOI:** 10.1002/ece3.2262

**Published:** 2016-07-19

**Authors:** Andrea M. Price, Vera Pospelova, Michael R. S. Coffin, James S. Latimer, Gail L. Chmura

**Affiliations:** ^1^ Department of GeographyMcGill University805 Sherbrooke Street WestMontréalQCH3A 0B9Canada; ^2^ School of Earth and Ocean SciencesUniversity of VictoriaOEASB A405PO Box 1700 STN CSCVictoriaBCV8W 2Y2Canada; ^3^ Department of BiologyUniversity of Prince Edward IslandCharlottetownPEIC1A 4P3Canada; ^4^ US Environmental Protection AgencyOffice of Research and DevelopmentNarragansettRhode Island02882USA

**Keywords:** Coastal waters, estuary type, northeast USA, palynology, phytoplankton, Prince Edward Island, water quality

## Abstract

Few biogeographic studies of dinoflagellate cysts include the near‐shore estuarine environment. We determine the effect of estuary type, biogeography, and water quality on the spatial distribution of organic‐walled dinoflagellate cysts from the Northeast USA (Maine to Delaware) and Canada (Prince Edward Island). A total of 69 surface sediment samples were collected from 27 estuaries, from sites with surface salinities >20. Dinoflagellate cysts were examined microscopically and compared to environmental parameters using multivariate ordination techniques. The spatial distribution of cyst taxa reflects biogeographic provinces established by other marine organisms, with Cape Cod separating the northern Acadian Province from the southern Virginian Province. Species such as *Lingulodinium machaerophorum* and *Polysphaeridinium zoharyi* were found almost exclusively in the Virginian Province, while others such as *Dubridinium* spp. and *Islandinium*? *cezare* were more abundant in the Acadian Province. Tidal range, sea surface temperature (SST), and sea surface salinity (SSS) are statistically significant parameters influencing cyst assemblages. Samples from the same type of estuary cluster together in canonical correspondence analysis when the estuaries are within the same biogeographic province. The large geographic extent of this study, encompassing four main estuary types (riverine, lagoon, coastal embayment, and fjord), allowed us to determine that the type of estuary has an important influence on cyst assemblages. Due to greater seasonal variations in SSTs and SSSs in estuaries compared to the open ocean, cyst assemblages show distinct latitudinal trends. The estuarine context is important for understanding present‐day species distribution, the factors controlling them, and to better predict how they may change in the future.

## Introduction

Marine biogeographic studies delineate distinct environmental regions of the ocean that are characterized by different species assemblages and have traditionally been carried out to explain the distribution of species, to understand the evolution of marine taxa, and to infer past climates and continental movement (Hedgepeth 1957; Hale 2010 and references within). Recent technical advances have resulted in more sophisticated marine biogeographic studies with the use of data from large‐scale observation programs, satellite imagery, and genetic techniques among others (Hale et al. 2013). Marine biogeographic classifications are important in ecosystem‐based management (McLeod and Leslie 2009), as they provide essential data for marine biodiversity conservation (e.g., Cook and Auster 2007) and protected areas (Spalding et al. 2007), and can be incorporated into climate change models that assess the effect of increased temperature on species distributions at different spatial scales (Hale et al. 2013).

Marine biogeographic classifications exist at different scales ranging dramatically in size from three (Ekman 1953) to over 230 (Spalding et al. 2007) subregions, and focus on different types of plants and animals such as fish (e.g., Briggs and Bowen 2012), ostracods (e.g., Hazel 1970), benthic macroinvertebrates (e.g., Hale 2010), and phytoplankton (e.g., Hasle 1976; Anderson et al. 1994; Okolodkov and Dodge 1996). However, few studies have documented biogeographic patterns of plankton in estuarine environments (e.g., Montoya‐Maya and Strydom 2009), despite the fact that many estuaries are experiencing negative effects of human (e.g., cultural eutrophication) and climate‐related stressors that may impact the distribution of plankton in coastal waters.

Phytoplankton are influenced by a variety of abiotic (e.g., temperature, salinity, nutrient loading) and biotic (e.g., grazing) factors. Phytoplankton blooms are related, in part, to water quality conditions and estuarine type. Estuary type is important because it incorporates many factors, such as tidal amplitude, freshwater input, turbidity, and water residence times. Estuary type can influence phytoplankton productivity and species assemblages in a variety of ways (e.g., Cloern et al. 1985). For example, river‐dominated estuaries may have high turbidity due to suspended particles, causing phytoplankton to be severely limited by light (e.g., Cloern et al. 1985). Physical mixing via tides, wave action, or other currents impacts water column stability and the degree of stratification. Some groups of phytoplankton, such as diatoms, thrive in well‐mixed waters, whereas others such as flagellates do well in more stratified waters (e.g., Huisman et al. 2004). Dinoflagellates are one group of plankton that may reflect estuary type (Pospelova et al. 2002).

Dinoflagellates are a major group of microplankton found in marine, brackish, and freshwater environments. Approximately half of species are autotrophic and are important for their contribution to primary production, while the other half are heterotrophic or mixotrophic (Dale 2009) and prey upon organisms such as diatoms, bacteria, and flagellates (Jacobson and Anderson 1986; Naustvoll 2000).

Although motile‐stage dinoflagellates are common in the plankton, they are rarely found in the sedimentary record as they are easily degraded. However, in coastal regions, numerous dinoflagellate species produce an organic‐walled resting cyst, a dormant stage that is resistant to biological, chemical, and physical degradation (e.g., Dale 1996). Once cysts are produced, they settle to the seafloor where they become incorporated into the sediment. Surface sediment samples represent an integrated assemblage of cyst production. The applicability of cysts as bioindicators of environmental conditions (e.g., sea surface temperature [SST], sea surface salinity [SSS], primary productivity, nutrient availability, and sea ice cover) is well known as the relationship between cyst assemblages and specific or specified above environmental parameters is commonly used for paleoenvironmental reconstructions (e.g., de Vernal et al. 1993, 2005; Dale 1996; Matsuoka 1999; Rochon et al. 1999; Ellegaard et al. 2006; Harland et al. 2006; Radi et al. 2007; Marret et al. 2008; Pospelova et al. 2015). Cysts produced by heterotrophic taxa increase in response to nutrient enrichment, due to increased prey availability, notably diatoms (e.g., Naustvoll 2000).

Dinoflagellate cysts from marine and coastal environments correspond to biogeographic zones (e.g., Dale 1996). Yet, biogeographic studies of dinoflagellate cysts (e.g., Wall et al. 1977; Dale 1996; de Vernal and Marret 2007) have included few samples from near‐shore estuarine environments. Estuaries are dynamic environments and experience greater variation in temperature, salinity, nutrients, mixing, and pollution than the fully open ocean environment due to riverine discharge, shallower water depths, and nutrient loading from continental sources. Dinoflagellate cysts are able to reflect water quality conditions at small spatial scales and thus are suitable for capturing the heterogeneity within an estuary (e.g., Pospelova et al. 2004, 2005; Pospelova and Kim 2010). Little is known about the distribution of cyst‐forming dinoflagellates at the estuarine scale, despite the common use of cysts as bioindicators. Providing a biogeographic and estuarine context will better enable researchers and managers to interpret dinoflagellate cysts as indicators of human‐ and climatic‐induced stressors.

In this study, we hypothesize that estuary type will be a major driver of dinoflagellate cyst assemblages and that the assemblages will differ according to biogeographic province. Different estuary types are characterized by specific geological and physical characteristics, which influence how environmental variables change within an estuary and their susceptibility to eutrophication. Because few studies have documented biogeographic province patterns in near‐shore estuarine environments (e.g., Wall et al. 1977), our study expands the knowledge of cysts and the plankton that produce them. We analyzed surface sediments from two biogeographic provinces (Acadian and Virginian) and four estuary types in the northwest Atlantic. The biogeographic classification scheme reviewed by Hazel et al. (1970) and used by Hale (2010) is used in this study and delineates the boundary between the Acadian and Virginian provinces at Cape Cod.

## Methods

### Study area

#### Estuary types

Estuaries have conventionally been classified by their geomorphic or hydrodynamic properties (e.g., Pritchard 1952; Dyer 1973). However for ecological purposes, neither is adequate to sufficiently characterize the range in estuarine features. For our study, the Coastal and Marine Ecological Classification Standard (CMECS) conceptual classification scheme was used to classify estuaries. The CMECS is based on a combination of geomorphological, energy, and biogeographic classifiers and divides estuaries into four dominant types: riverine, lagoon, coastal embayment, and fjord (Glibert et al. 2010). Riverine estuaries tend to be highly flushed with a large salinity range and greater seasonal stratification. They are often characterized by a V‐shaped channel and a salt wedge. Coastal lagoons are generally shallow and enclosed, with low flushing rates and little current action. Due to their shallow water depths, they can also be wind‐mixed. Coastal embayments are estuaries that are loosely bounded by landforms and are open to greater exchange with the ocean. They often have moderate to high salinities, are well flushed, and can receive high‐energy inputs. In this classification scheme, fjords are deep estuaries, with low to moderate river input, experience some stratification, and may have a sill at the mouth of the estuary.

**Figure 1:**
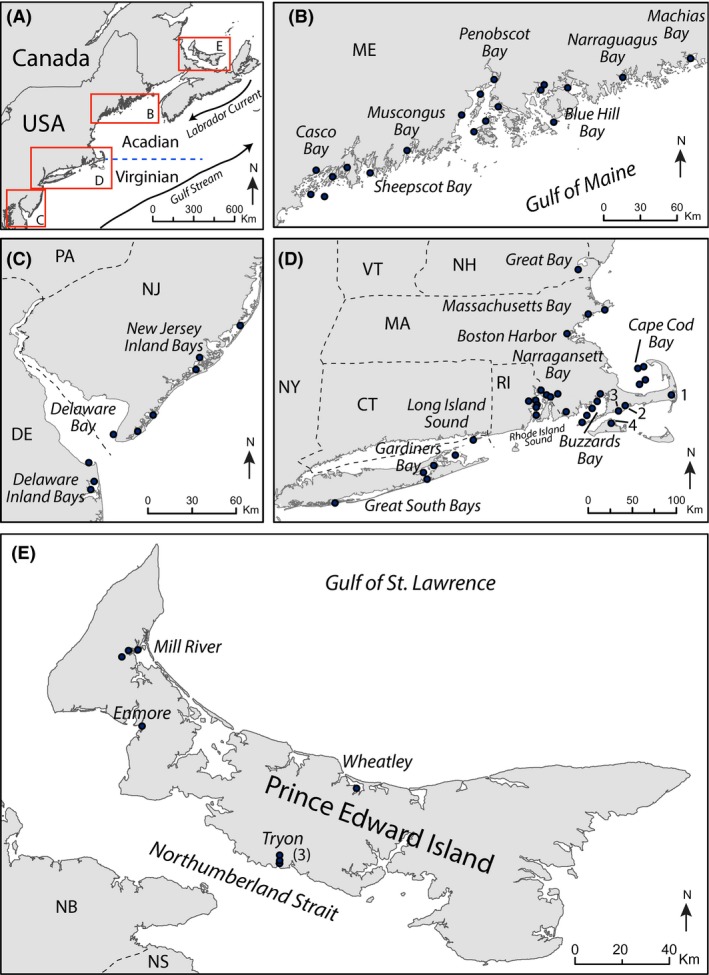
(A) The northeast coast of the USA and Canada. The dashed line indicates the boundary between the Acadian and Virginian biogeographic provinces. Arrows show the direction of Labrador Current and the Gulf Stream. (B–E) Insets of the study area. Solid dots indicate sampling locations. (D) 1. Chatham Harbor. 2. Popponesset Bay. 3. Waquoit Bay. 4. Vineyard Pond.

Our study examined 23 estuaries from Maine to Delaware in the northeast USA and four estuaries in Prince Edward Island, Canada (Fig. 1). Estuaries vary according to their glacial history and bedrock geology, wave and tidal action, and sediment supply. Of the studied estuaries, seven are fjords, 12 are coastal embayments, three are river dominated, and five are lagoons.

Mill River and Wheatley estuaries are located on the north shore of PEI (Gulf of St. Lawrence) where a high supply of sediment and a microtidal range (<1 m) result in the formation of spits and barrier islands. Conversely, Enmore and Tryon estuaries located on the south shore of PEI (Northumberland Strait) are mesotidal (tidal range ~ 3 m) and are coastal embayments.

Northern Maine, from Casco Bay to Machias Bay, is characterized by fjord‐type estuaries and has some of the highest tidal ranges (2.7–3.8 m) in the study area. These estuaries are generally deep (average depth ~ 10–22 m) reflecting their glacial history; however, many are relatively wide and do not have a sill. The dominant factor controlling circulation patterns in these estuaries is tidal mixing, due to low freshwater input and large tidal ranges (Roman et al. 2000). Water clarity is typically higher in these fjord‐type estuaries, compared to southern coastal plain estuaries owing to low river flows, small‐forested watersheds, bedrock substrate, and low sediment loads (Roman et al. 2000).

From southern Maine to Cape Cod (MA), the most common estuary type is coastal embayment and the tidal range is mesotidal (2.4–2.9 m). Southwest of Cape Cod, along the coast of Rhode Island and Connecticut, many of the estuaries are orientated north–south and were originally formed as coastal plain sediments were eroded (Roman et al. 2000). Coastal embayment or riverine‐type estuaries are common, along with lagoons. The tidal range is microtidal (0.3–1.2 m), and the average depth is <10 m. Further south, many of the estuaries in New York, New Jersey, and Delaware are located behind barrier islands (e.g., Great South Bay, the New Jersey Inland Bays). These lagoon‐type estuaries are very shallow, with average depths of 1–2 m, and are microtidal (0.2–1 m tidal range).

#### Sea surface temperatures and hydrodynamics

A strong thermal boundary at Cape Cod (MA) has a major control on species distribution. The Acadian Province, north of Cape Cod, is generally cooler and experiences a smaller seasonal range in SSTs. Winter temperatures are similar in both areas (~2–4°C), but in summer the Virginian Province, south of Cape Cod, is warmer (>22°C) than the Acadian Province (<~20°C) due to latitudinal differences in solar radiation (e.g., Loder et al. 1998). However, many estuaries in PEI are shallow (<5 m) and also experience SSTs >20°C in summer (e.g., Shaw 1999). In winter, PEI estuaries are covered by up to 1.2 m of ice. Sea ice can also form for brief periods during cold winters on the northeast US coast and can be found as far south as Rhode Island Sound (Fig. 1D). Oceanographic currents also influence estuarine temperatures, salinity, and nutrient concentrations. Perhaps the most important of these is the Labrador Current (Fig. 1A), where one branch flows southwestward along the continental shelf carrying cold, relatively freshwater.

The primary sources of freshwater to the northeast coast are continental runoff from local rivers and streams, and the coastal limb of the Labrador Current (Loder et al. 1998; Townsend et al. 2006). These reduce salinity, promote thermohaline stratification, and can provide a substantial input of nutrients. In coastal regions of the Gulf of Maine (Fig. 1B), strong tidal mixing prevents stratification from developing. Mixing brings nutrients from depth up into the euphotic zone and promotes primary productivity. In the northern reaches of the Virginian Province, primary productivity is fueled by tidal mixing of nutrient‐rich deep waters and winter convective mixing which supplies nutrient‐rich waters to the surface. Winter–spring phytoplankton blooms often occur early in the year when the SSTs are still cool. In warmer months, the water column often experiences greater stratification, encouraged by freshwater inputs and warmer SSTs. Shallow areas with strong tidal currents, on the other hand, may remain unstratified (Garrett et al. 1978).

### Sediment collection

Northeast USA surface sediments were collected using a 0.04‐m^2^ Young‐modified Van‐Veen grab or similar sampler, by the US Environmental Protection Agency (EPA) in 2000 (Strobel 2000). Within an estuary, the sampling locations were randomly selected using the Environmental Monitoring and Assessment Program's probabilistic sampling framework, and range from the head to the estuary mouth. One sediment sample was collected at each site over the course of July through October 2000 (EPA 2006). A total of 61 samples were selected from sites with a summer SSS > 20 (Table 1). An additional eight PEI sediment samples were collected using an Ekman dredge in summer of 2013, also with surface salinities >20 (Table 1). At all sites, the top 2 cm of sediment was retained for analysis and represents approximately 6–20 years of accumulation (estimated sedimentation rate of ~0.1–0.3 cm·year^−1^) (Fitzgerald 1980; Sonshine 2012). After the sediment was homogenized, subsamples were taken for grain size and geochemical analyses. The number of samples per estuary ranges from one to nine.

**Table 1:** Sampling locations together with sedimentary (% clay and % biogenic silica) and average summer sea surface hydrographic (sea surface temperature and sea surface salinity) characteristics. EDA is the estuary drainage area code, and CMECS is the Coastal and Marine Ecological Classification Standard

Province/State	Estuary name	EDA	CMECS estuary type	ID #	Location			Sediment		Hydrography	
					Station depth (m)	Latitude (°N)	Longitude (°W)	Clay (%)	BSi (%)	SST (°C)	SSS (psu)
PEI	Tryon		Coastal embayment	2963	2	46.232	−63.541	41.4	7.9	21.1	22.9
				2964	2.5	46.213	−63.539	35.2	5.9	20.4	27.0
				2965	2.3	46.204	−63.536	3.6	4.6	18.6	28.9
	Mill River		Lagoon	2972	1.5	46.747	−64.166	68.3	5.0	20.2	22.0
				2973	3.6	46.772	−64.119	66.0	6.4	20.2	22.9
				2974	2.9	46.768	−64.072	4.5	8.1	19.2	25.8
	Wheatley		Lagoon	2980	4.5	46.415	−63.233	78.4	4.1	20.6	27.1
	Enmore		Coastal embayment	2994	2.1	46.576	−64.062	18.1	10.1	21.0	25.2
ME	Machias Bay	N020x	Fjord	06‐393	14.0	44.669	−67.346	77.6	4.2	11.4	N/A
	Narraguagus Bay	N030x	Fjord	06‐392	4.8	44.528	−67.844	18.1	7.5	12.7	32.1
	Blue Hill Bay	N040x	Fjord	06‐390	37.8	44.449	−68.249	69.6	10.2	15.0	33.1
				06‐386	2.0	44.472	−68.423	44.5	4.6	19.3	24.3
				06‐384	23.6	44.434	−68.447	93.8	11.6	18.8	27.1
				06‐382	10.9	44.192	−68.352	8.2	15.7	14.6	31.5
	Penobscot Bay	N050x	Fjord	06‐385	20.0	44.511	−68.790	42.1	5.4	16.1	17.1
				06‐383	17.7	44.403	−68.891	80.9	8.7	13.3	29.3
				06‐381	24.0	44.307	−68.760	87.0	9.8	14.1	29.3
				06‐380	13.3	44.247	−69.029	22.4	9.4	16.2	25.0
				06‐379	17.2	44.201	−68.852	73.5	8.8	12.6	30.3
				06‐378	7.4	44.120	−68.937	4.4	3.8	14.3	29.7
	Muscongus Bay	N060x	Fjord	06‐388	5.2	43.980	−69.429	72.0	12.5	15.4	32.4
	Sheepscot Bay	N080x	Fjord	06‐387	46.8	43.813	−69.700	58.6	8.4	13.6	31.6
	Casco Bay	N100x	Fjord	06‐377	2.5	43.853	−69.868	77.0	9.0	18.9	31.0
				06‐375	14.2	43.784	−69.975	55.9	9.7	15.7	31.0
				06‐376	1.5	43.832	−70.097	31.0	12.3	18.9	27.3
				06‐373	47.7	43.636	−70.036	9.5	12.1	14.8	29.8
				06‐374	36.7	43.652	−70.136	51.1	6.6	N/A	N/A
NH	Great Bay	N130x	Riverine	06‐343	1.8	43.001	−70.939	60.9	6.0	21.5	21.2
MA	Boston Harbor	N170a	Coastal embayment	06‐371	11.2	42.348	−71.054	N/A	7.4	18.1	28.1
	Massachusetts Bay	N170w	Coastal embayment	06‐355	6.4	42.590	−70.665	4.7	6.6	17.4	30.5
				06‐354	10.3	42.548	−70.835	22.6	9.7	15.2	29.5
	Cape Cod Bay	N180x	Coastal embayment	06‐360	46.1	42.011	−70.270	32.8	8.9	18.6	31.0
				06‐359	47.1	41.993	−70.329	85.4	6.1	18.6	31.0
				06‐358	30.3	41.877	−70.253	32.3	8.4	19.1	30.8
				06‐357	25.4	41.830	−70.313	26.7	7.3	18.9	31.0
	Chatham Harbor	N/A	Coastal embayment	06‐370	4.4	41.720	−69.986	65.0	7.6	N/A	N/A
	Popponesset Bay	N/A	Coastal embayment	06‐369	0.7	41.613	−70.457	66.9	11.6	N/A	N/A
	Vineyard Ponds	N/A	Coastal embayment	06‐367	8.0	41.433	−70.602	43.2	13.0	N/A	N/A
	Waquoit Bay	N190x	Coastal embayment	06‐368	2.2	41.557	−70.526	35.0	4.0	22.0	29.4
	Buzzards Bay	M010x	Coastal embayment	06‐348	2.1	41.734	−70.712	N/A	2.0	24.0	25.1
				06‐347	5.3	41.656	−70.744	4.6	14.0	23.4	30.3
				06‐346	11.1	41.584	−70.795	N/A	5.3	22.1	30.7
				06‐345	17.5	41.513	−70.848	59.0	6.2	21.3	31.2
				06‐344	13.3	41.442	−70.899	N/A	2.5	21.8	30.7
				06‐363	1.5	41.551	−71.061	36.5	17.1	24.7	26.2
RI	Narragansett Bay	M020x	Riverine	06‐420	1.5	41.771	−71.319	55.9	5.4	N/A	N/A
				06‐419	0.9	41.722	−71.262	53.4	6.7	N/A	N/A
				06‐418	12.5	41.732	−71.146	43.5	6.0	N/A	N/A
				06‐416	5.0	41.701	−71.220	71.0	7.9	22.7	27.5
				06‐415	7.0	41.667	−71.372	12.6	10.2	21.8	28.7
				06‐412	3.5	41.659	−71.444	53.6	12.7	23.1	28.1
				06‐413	8.0	41.621	−71.358	54.3	6.3	21.4	29.0
				06‐409	8.2	41.597	−71.366	74.2	6.4	21.1	29.4
				06‐408	5.5	41.515	−71.366	11.3	7.0	19.6	30.4
NY	Gardiners Bay	M030x	Coastal embayment	06‐407	9.6	41.108	−72.193	58.5	4.5	21.2	29.1
				06‐404	9.5	41.000	−72.412	61.6	6.1	22.6	28.3
				06‐403	6.1	40.931	−72.518	93.7	9.1	22.9	28.1
	Long Island Sound	M040w	Coastal embayment	06‐421	3.5	41.262	−72.009	58.5	7.9	12.6	N/A
	Great South Bay	M050x	Lagoon	06‐402	2.4	40.860	−72.481	N/A	8.5	20.7	29.8
				06‐401	1.7	40.618	−73.421	64.2	7.3	21.8	30.6
NJ	New Jersey Inland	M080x	Lagoon	06‐398	2.1	39.640	−74.205	55.4	6.4	22.6	28.5
	Bays			06‐397	0.9	39.444	−74.454	87.1	9.9	23.2	30.3
				06‐396	2.1	39.370	−74.477	34.8	4.5	22.8	30.0
				06‐395	6.2	39.091	−74.739	75.3	12.4	21.0	32.1
				06‐394	0.6	38.992	−74.833	28.0	3.3	20.9	32.5
NJ and DE	Delaware Bay	M090x	Riverine	06‐399	7.6	38.972	−74.982	16.8	7.1	22.0	26.2
				06‐353	6.0	38.800	−75.134	32.9	10.7	22.5	26.9
DE	Delaware Inland	M100x	Lagoon	06‐352	1.9	38.685	−75.101	69.3	6.1	23.8	29.0
	Bays			06‐350	1.9	38.635	−75.121	30.9	9.6	23.0	29.2

### Palynological sample preparation and microscopy

Sediments were treated using a standard palynological method as described by Pospelova et al. (2005). First, a known volume of sediment was rinsed with distilled water, oven‐dried at 40°C, and weighed. One tablet of exotic marker grain *Lycopodium clavatum* was added prior to sample treatment to enable the determination of cyst concentrations. The samples were treated with 10% hydrochloric acid and 48% hydrofluoric acid to remove carbonates and silicates, and sieved at 15 and 125 *μ*m to remove both coarse and fine fractions. Oxidation agents were avoided to prevent the loss of delicate dinoflagellate cysts (e.g., Zonneveld et al. 2010). Aliquots of residue were mounted in glycerin jelly, and cysts were examined at 400–1000× magnifications. A minimum of 118 cysts were counted in each sample, with an average of 346. Cyst concentrations were calculated using the dry weight of the sample. Palynological slides are stored at the Paleoenvironmental Laboratory, SEOS, University of Victoria, Canada.

The species name of the motile stages is not known for most of the dinoflagellate cyst species found in this study; therefore, the paleontological nomenclature is used. Dinoflagellate cysts were identified according to the taxonomic system described by Lentin and Williams (1993), Rochon et al. (1999), and Zonneveld and Pospelova (2015). Cysts were identified to the species level where possible. A list of grouped taxa and the detailed descriptions of cyst types can be found in Appendix S1. A table with all cyst taxa recorded in this study and their corresponding motile stage is found in Table S1 in Appendix S2.

### Hydrographic and sediment chemistry data

Long‐term measurements of US hydrographic variables were obtained from sites nearby the sediment sampling location and represent the estimated duration of sediment accumulation. Measurements of summer (June–September) SST and SSS were averaged for use in statistical analyses (Table 1). Data from other seasons were not consistently collected and thus not used in this study. Grain size analysis was completed by the EPA through its Environmental Monitoring and Assessment Program (http://www.epa.gov/emap/) and follows the methodology of Strobel et al. (1995) where the sediment was homogenized, diluted with water, and passed over a 63‐*μ*m sieve. Both the fine and coarse fractions were retained, dried, and weighed. Particles smaller than 63 *μ*m were considered silt‐clay, while those larger than 63 *μ*m were defined as sand.

Sea surface salinity and SST values from PEI estuaries were collected approximately biweekly over the course of one summer using a YSI V2 6600 multiparameter sonde (Yellow Springs, OH). Data from other seasons are sparse and were not used. Grain size analysis on PEI sediments was completed using the same method as the EPA, with the exception that no chemical dispersant was added.

Biogenic silica content for all samples was analyzed at the Pacific Center for Isotopic and Geochemical Research (PCIGR) at the University of British Columbia, Canada. The standard wet alkaline procedure was used (Mortlock and Froelich 1989). Percent biogenic silica is calculated using the following equation: % opal = 2.4 × % Si_OPAL_ (Mortlock and Froelich 1989), which takes into account a correction for a relatively constant water content of ~10% in diatomaceous silica.

### Statistical analyses

Multivariate analyses were performed on dinoflagellate cyst relative abundance and environmental data using CANOCO 4.0 (ter Braak and Šmilauer 2002). First, the nature of variability within the cyst assemblage was tested using Detrended Correspondence Analysis (DCA). The length of the first gradient was 3.0 standard deviations, indicating unimodal variation and justifying the use of Canonical Correspondence Analysis (CCA). Variables that are statistically significant (*P*‐value < 0.05) in explaining variation in the cyst assemblage were determined using a forward selection Monte Carlo permutation test, with 499 unrestricted permutations. Environmental variables included in the analysis are tidal range, water depth, % clay, % biogenic silica, and average summer SST and SSS. An adjusted explained variation ($R_{\mathrm{adj}}^{2}$) was calculated according to Peres‐Neto et al. (2006) using the formula $R_{\mathrm{adj}}^{2}$ = 1 − ((*n *− 1)/(*n *− *p *− 1)) × (1* *− *R* ^2^), where *n* is the number of samples and *p* is the number of predictors or explanatory variables. Principal Components Analysis (PCA), an unconstrained ordination method, was also performed on relative abundance data.

## Results

**Figure 2:**
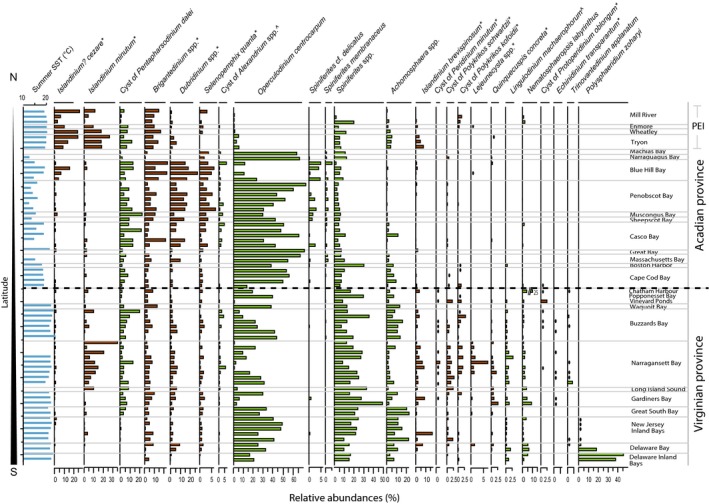
Summer sea surface temperature (SST) and relative abundance of selected taxa. Samples are ordered by latitude (north to south), and the dashed line across the figure separates the Acadian Province from the Virginian Province. Brown bars and * denote heterotrophic taxa, green bars represent autotrophic taxa, and ^ denotes toxic taxa.

Many dinoflagellate cyst taxa showed spatially restricted distribution patterns (Fig. 2, Figs. S1–S8 in Appendix S2) that reflect biogeographic provinces demonstrated by other marine organisms. In general, the Acadian Province has greater relative and absolute abundance of *Spiniferites* cf. *delicatus*,* S. membranaceus*,* Islandinium*? *cezare*, and *Dubridinium* spp. (Fig. 2, Fig. S2 in Appendix S2). The cosmopolitan species *Operculodinium centrocarpum* is one of the dominant taxa in Acadian estuaries (Fig. S3 in Appendix S2), where it comprises between 30% and 70% of assemblages, with the exception of sites in PEI and Blue Hill Bay (ME). Blue Hill Bay has a greater proportion of heterotrophic taxa (44–70%) (Fig. S1 in Appendix S2) compared to the average of 21% in the rest of the study area, with *Brigantedinium* spp., *Dubridinium* spp., and *I. cezare* accounting for most of this increase.

The Virginian Province has higher abundance of *Spiniferites* spp., *Achomosphaera* spp., spiny brown cysts, and cysts of *Polykrikos* (Fig. 2, Figs. S3, S5–S7 in Appendix S2). This province also has greater abundance of cyst of *Peridinium minutum* sensu Wall and Dale 1968, cyst of *Protoperidinium oblongum*,* Echinidinium transparantum*,* Trinovantedinium applanatum*, and *Polysphaeridium zoharyi* (Fig. 2, Figs. S7 and S8 in Appendix S2) compared to the Acadian Province. *Islandinium minutum* and *Lejeunecysta* taxa are common in Narragansett Bay (RI) (Fig. 2, Figs. S5 and S6 in Appendix S2), and *P. zoharyi* is only south of Long Island Sound where it comprises up to 46% (Fig. 2, Fig. S7 in Appendix S2).

**Figure 3:**
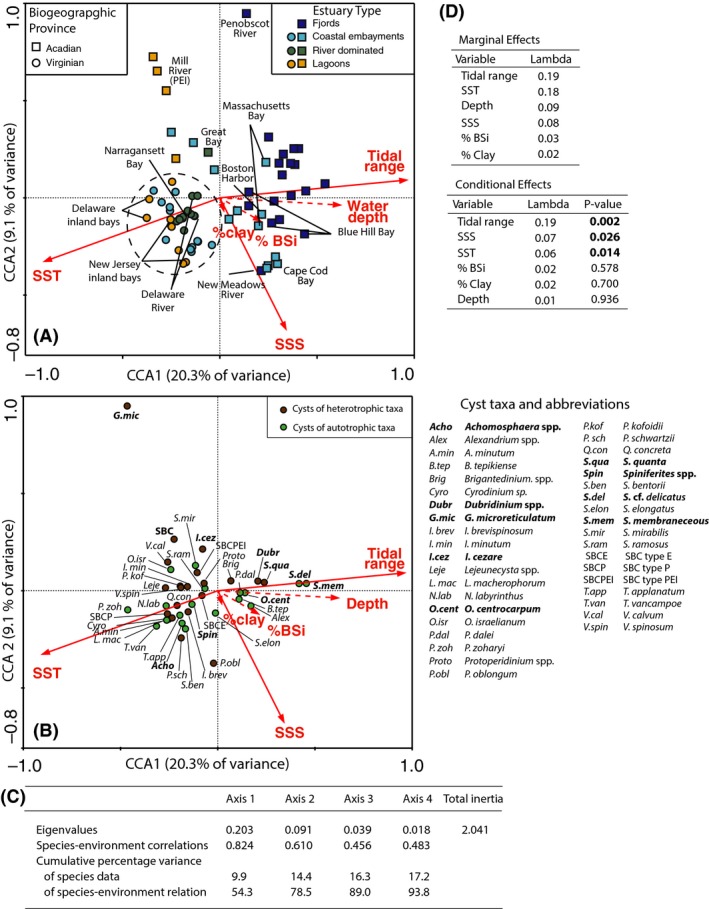
Canonical Correspondence Analysis (CCA) ordination diagrams of dinoflagellate cyst relative abundance and environmental variables. Statistically significant variables (*P *< 0.05), environment variables are shown with solid arrows. (A) Sample scores, where different colors and symbols differentiate between estuary type and biogeographic provinces. Samples from the Virginian Province cluster together as depicted by the dashed oval (B). Species scores. Species with the greatest best fit as a fraction of species variance for axes one and two are highlighted in boldface. (C) Eigenvalues for CCA axes 1–4, species environment correlations, and cumulative percentage of variance. (D) CCA marginal and conditional effects for environmental variables (tidal range, sea surface temperature (SST), water depth, sea surface salinity (SSS), % biogenic silica (%BSi), and % clay). Marginal or simple effects summarize the effects of each predictor variable individually. Conditional effects summarize the partial effect of each predictor variable, representing the variation explained by the predictor variable after accounting for the effect of the variables above it in the list (ter Braak and Šmilauer 2012).

Of the six environmental variables used in the CCA tidal range, SST and SSS were statistically significant (Fig. 3D). The first axis correlates with SSTs and tidal range, while the second axis correlates with SSSs. The first two CCA axes have eigenvalues of 0.203 and 0.091 and explain 54.3% and 24.2% of the species–environment relation, respectively (Fig. 3C). The total variation explained by the environmental variables after accounting for covariate effects is 20.2%, while the adjusted explained variation is 12.0%. Samples from the Virginian Province tightly cluster together, having negative CCA1 scores and generally negative CCA2 scores. On the other hand, samples from the Acadian Province are spread throughout the diagram, but do not overlap with samples from the Virginian Province (Fig. 3A). In the PCA, samples from the two biogeographic provinces can be distinguished, but estuary type is less evident.

Samples from the same estuary type cluster together when from the same biogeographic province. Fjord‐type estuaries are clustered on the right‐hand side of the diagram toward higher tidal range, greater estuary depth, and low summer SSTs. As a whole, samples from coastal embayments are not tightly clustered together, but instead appear to be separated based upon their biogeographic province. Coastal embayments in the Acadian zone are ordinated closer to the samples from fjord‐type estuaries, compared to those from the Virginian zone. River‐dominated estuaries have negative CCA1 scores and correlate with warmer SSTs, lower tidal ranges, and shallower water depths. Great Bay is the only river‐dominated estuary in the Acadian Province and has a more positive CCA2 score than those from the Virginian that are clustered tightly together (Fig. 3A). Samples from the lagoons are all ordinated on the left‐hand side of the diagram and correlate with warmer SSTs, low tidal ranges, and low water depths. They also can be differentiated based on the biogeographic province. In this study, the only lagoons in the Acadian Province are from PEI and they have very positive CCA2 scores and correlate with low salinity. Lagoons in the Virginian Province, on the other hand, have more negative CCA1 and CCA2 scores and correlate with high SSTs and SSSs and low tidal ranges.

Some taxa such as *P. zoharyi*,* Tuberculodinium vancampoae*,* Lingulodinium machaerophorum, Cyrodinium* sp., and cyst of *P. minutum* sensu Wall and Dale 1968 correlated with warm SSTs (Fig. 3B). Conversely, *S*. cf. *delicatus*,* S. membraneceous, Selenopemphix quanta*, and *Dubridinium* spp. correlate with cooler summer SSTs. Cyst of *Gymnodinium microreticulatum* is an outlier with a very high CCA2 score and correlates with lower summer SSSs and warmer summer SSTs (Fig. 3B). The best fit as a fraction of species variance for CCA1 is *S. quanta* (38%), *S. membranecous* (32%), *Dubridinium* spp. (28%), *O. centrocarpum* (26%), *Spiniferites* spp. (25%), *S*. cf. *delicatus* (21%), and *Achomosphaera* spp. (29%). For CCA2 cyst of *G. microreticulatum* (19%), *Achomosphaera* spp. (17%), SBCs (17%), and *I. cezare* (9%) have the greatest fit (Fig. 3B).

## Discussion

With the exception of Wall et al. (1977), previous estuarine dinoflagellate cyst studies in North America have been geographically limited, generally pertaining to one bay (Verardo 1999), relatively small (<150 km) spans of coastline (e.g., Godhe and McQuoid 2003; Pospelova et al. 2004, 2005), or including only one estuary type (e.g., Richerol et al. 2012). Here, we show the response of dinoflagellate cysts to SST, SSS, and estuarine type over an extensive (>1000 km) geographic area. Both cyst relative abundance and absolute abundance (concentrations) show trends similar to one another.

### Estuarine biogeography

The predominant pattern observed in the cyst record in the northwest Atlantic is the differentiation between the Acadian and Virginian biogeographic provinces. The Acadian Province is generally cooler, deeper, more saline and has a greater tidal range than those south of Cape Cod (Table 1). Multivariate ordination of both dinoflagellate cysts (Fig. 3) and subtidal benthic macroinvertebrates (Hale 2010) from northwest Atlantic estuaries shows two distinct clusters, where samples from the Acadian Province are separate from those in the Virginian Province and lend support to the validity of the two biogeographic provinces. Further, using the classification scheme by Spalding et al. (2007), the cyst assemblages would support the boundary between the Virginian and Gulf of Maine/Bay of Fundy subregions, also at Cape Cod. This shows the broad application of these biogeographic provinces, as even short‐lived plankton demonstrate distinct distribution patterns.

The Acadian Province is characterized by the higher abundance of *I*. *cezare*,* Dubridinium* spp., *S. quanta*, cysts of *P. dalei* and *I. minutum*. Along the northeast coast of Canada in Nunatsiavut (Labrador), Richerol et al. (2012) also document high abundance of cysts of *P. dalei* (25–71%) in Okak and Anaktalak fjords, relatively high abundance of *I. minutum* (34–58%), and moderate abundance of *I. cezare* (5–11%) in more northerly Nachvak and Saglek fjords. Richerol et al. (2012) relate the abundance of the heterotrophic taxa (i.e., *Brigantedinium* spp., *Islandinium* spp.) to increases in diatoms (prey availability) and to a lesser extent to sea ice and nutrients. *I. minutum, I. cezare*, and *P. dalei* may also prefer the cooler temperatures in the Acadian Province. *I*. *cezare* is found in highest abundance in subpolar to polar waters in the Northern Hemisphere, in particular areas where SSS is seasonally reduced due to melting snow and ice (Zonneveld et al. 2013). However, a recent study of dinoflagellate cysts from the Hudson Bay system (Heikkilä et al. 2014) has found that this species was associated with the highest fluxes of sedimentary biogenic silica – a proxy for diatom productivity. In our study area, *I*. *cezare* reaches highest abundance (24–26%) in PEI estuaries, at sites <5 m deep and at the head of Blue Hill Bay (16%) (Fig. 2), although it does not appear to be related to higher abundance of % biogenic silica. This lack of correlation may be because we do not have biogenic silica flux data only relative abundance data. We suspect that we would have found a significant correlation if our data included fluxes of biogenic silica and were not restricted to percentages. Thus, biogenic silica is not a significant factor in the multivariate ordination (Fig. 3D). In addition, although winter data were not available, it is very likely that winter SSTs are quite cool and that these sites experience more extended sea ice cover. *Dubridinium* spp. and *S. quanta* are common indicators of nutrient enrichment and high‐productivity coastal areas (e.g., Pospelova et al. 2002, 2005) and may reflect nutrient availability in the cooler Acadian Province.

Previous studies have suggested that thermal discontinuities at Cape Cod are the predominate cause for the biogeographic boundary, rather than the Cape acting as a physical boundary (e.g., Franz and Merrill 1980). Wares (2002) notes that more species reach their northern limit at the Cape, than those reaching their southernmost limit. This is also true for cyst‐producing dinoflagellates in our study, as more species reach their northernmost limit area at the Cape (e.g., *T. applanatum*, cyst of *P. oblongum*). This in part may be a result of the southward‐flowing Labrador Current, which is the predominant current along the coast, hindering the northward transport of plankton, or cooler SSTs experienced in the Acadian Province.

The most prominent characteristic that distinguishes the Virginian Province from the Acadian Province is the composition of cyst assemblages. In addition, a few taxa are not found or only rarely found in the Acadian zone (heterotrophic taxa – *Q. concreta*,* E. transparantum*,* T. applanatum*,* Cryodinium* sp.; autotrophic taxa – *L. macaerophorum* and *P. zoharyi*) (Fig. 2, Figs. S6–S8 in Appendix S2). Of these, only *P. zoharyi* comprises a significant proportion of the assemblages. Both *L. macaerophorum* and *P. zoharyi* are recognized as warm water taxa and reported in high abundance in temperate to tropical, near‐shore environments (e.g., Zonneveld et al. 2013). The heterotrophic taxa that have rarely been found north of Cape Cod (*Q. concreta*,* E. transparantum*,* T. applanatum*,* Cryodinium* sp.) make up a minor component of the assemblages in the Virginian Province, and their low abundance suggests that they have a narrower ecological niche. For instance, they may only thrive in a particular season or have more selective prey preferences. These heterotrophic taxa have been recorded elsewhere in even cooler waters, so the warmer waters in the Virginian Province are not the only factor accounting for their presence in this province. Instead, the large range in seasonal temperatures in the Virginian Province may be more favorable, or the presence of particular food sources for cyst‐producing heterotrophic dinoflagellates.

In the Virginian Province, there is a distinct increase in *Spiniferites* spp. and *Achomosphaera* spp. *Spiniferites* taxa are common in estuaries (e.g., Ellegaard 2000; Pospelova et al. 2004, 2005; Price and Pospelova 2011) and are able to thrive in regions with large salinity variations (Pospelova et al. 2002, 2005). Their increase in the Virginian Province does not appear to be directly linked to a decrease in salinity, but perhaps is related to a moderate increase in summer temperature as these taxa show some correlation with summer SSTs (Fig. 3). Alternatively, they may be better able to tolerate the large seasonal fluctuations seen in the Virginian Province, as they may have an intrinsically broader niche.

As neither autotrophic nor heterotrophic taxa display any discernible pattern with respect to the ordination axes (Fig. 3B), we conclude that dinoflagellate trophic level does not play a major role in separating biogeographic provinces or estuary types. This is not surprising as the ratio of heterotrophic to autotrophic taxa is often associated with nutrient/prey availability, with higher ratios occurring with increasing eutrophication or in regions of higher productivity (e.g., Harland 1973; Lewis et al. 1990; Matsuoka 1999). Eutrophication is not related to geography, but primarily to anthropogenic influence, and as such, nutrient loading is likely a greater contributor to trophic‐level structure.

Wall et al. (1977) discuss the importance of hydrodynamic boundaries as key controls on cyst biogeography and suggest the presence of two important types of hydrodynamic boundaries: (1) between estuarine and coastal (shelf) waters and (2) between coastal and oceanic water. Across shelf transects in their study allowed them to identify these onshore–offshore patterns. Ours is the first study of entirely estuarine environments and we identify a strong latitudinal zonation, in contrast to Wall et al. (1977) who observed only a weak latitudinal zonation in the North and South Atlantic. Because near‐shore environments are more likely to show better latitudinal trends (Wall et al. 1977) due to greater seasonal variations in SSTs and SSSs, it may explain our differences in the importance of latitudinal trends. Furthermore, dinoflagellate cyst assemblages from the open ocean may contain a large proportion of transported cysts (e.g., Dale 1996), thereby diluting the climatic signal from the overlying water column.

### Estuary‐type signal

Pospelova et al. (2002) proposed that estuary type influences cyst assemblages and highlighted a need for an extensive study of dinoflagellate cysts in a wide range of estuaries. There are few dinoflagellate cyst studies in estuaries, and most previous studies have focused only on one or two estuary types or a narrow latitudinal range (<0.5°) (e.g., Pospelova et al. 2004, 2005; Richerol et al. 2012). This study purposely encompasses all four estuary types (fjord, coastal embayments, riverine, and lagoons); therefore, the effect of estuary type on the cysts signal can be identified. If estuary type had no effect, we would expect samples from the same type to show no clustering in our dataset. Instead, we see samples from the same estuary type cluster together in CCA (Fig. 3A). While the four estuary types are not evenly distributed between the two zones, both estuary type and biogeography can be distinguished. River‐dominated estuaries show the tightest degree of clustering in CCA (Fig. 3), where salinities are brackish and in our study SSTs are moderate to warm. Fjord‐type estuaries have lower SSTs, a greater water depth, and higher tidal ranges. They are characterized by higher abundances of *O. centrocarpum*,* Dubridinium* spp., and *S. quanta*. Coastal embayments are variable in terms of their environmental properties as well as dinoflagellate cyst species assemblages. Lagoons are often very shallow, have barriers (islands or spits) limiting exchange with the open ocean, and can be wind‐mixed. As such, they can experience very warm summer SSTs, even at mid‐latitudes. Lagoons in the northwest Atlantic are characterized by higher abundance of warm water taxa including *T. vancampoe, L. machaerophorum*, and in PEI cysts of *G*. *microreticulatum*. Tight clusters are not observed for all estuary types, but can be partially explained by intraestuary variability. Samples from the mouth differ from those at the head of the estuary, due to the differences in nutrients, mixing, salinity, and temperature.

### Interestuary variability

Some estuaries have distinct assemblages that set them apart from other estuaries in the study area. Blue Hill Bay (ME) stands out from other estuaries in the Acadian Province due to its very low abundance of *O. centrocarpum* and high abundance of the heterotrophic taxa *Brigantedinium* spp., *Dubridinium* spp. and *I. cezare* (Fig. 2). This bay is partially stratified in the summer and well mixed the remainder of the year. The greater abundance of these heterotrophic taxa may suggest that this estuary is more susceptible to nutrient enrichment. Sites from Blue Hill Bay also correlate with high % biogenic silica values and high tidal range the CCA diagram (Fig. 3), suggesting that tidal mixing and perhaps an abundant food supply (diatoms) are present.

In the Virginian Province, Narragansett Bay (RI) has one of the most distinct assemblages due to the high abundance of *I. minutum* and other spiny brown cysts, as well as the consistent presence, albeit low abundance of *Lejeunecysta* and *Polykrikos* species (Fig. 2, Figs. S5 and S6 in Appendix S2). In Narragansett Bay, *I. minutum* is likely present in winter to early spring when SSTs are still very low and when diatoms are the dominant component of the phytoplankton assemblage from December to April (Pratt 1959; Oviatt et al. 2002). *Islandinium minutum* is not often found in such high abundance this far south; however, Smayda (1973) also record the presence of boreal–arctic plankton species in Narragansett Bay. *Islandinium minutum* is commonly known as a sea ice indicator (e.g., Potvin et al. 2013; de Vernal et al. 2013) and is found from temperate to polar regions, with high relative abundance in regions that experience large ranges in seasonal contrasts (Zonneveld et al. 2013). However, in a seasonal sediment trap study in Hudson Bay (Canada), *I. minutum* was produced in open water during a polar summer together with other heterotrophic taxa (Heikkilä et al. 2016). In the Hudson Strait, it reached its highest abundance in areas coinciding with the highest values of %BSi (Heikkilä et al. 2014), suggesting that prey availability is an important factor influencing the distribution of this species.

Within Narragansett Bay, we observed highest abundance (15–35%) of *I. minutum* in the uppermost reaches of the bay where high seasonal fluctuations in temperature (−1 to +25°C, Smayda 1973) and salinity (8–31, Oviatt et al. 2002) are also encountered. This is in agreement with other estuarine studies, such as Pospelova et al. (2004) where highest abundance (7.5%) of *I. minutum* is recorded at the head of Winnapaug Lagoon where winter SSTs are ~1°C and summer SSTs are ~22°C. The bay is also known for high levels of primary production (e.g., Oviatt et al. 2002) compared to surrounding New England coastal waters (Pratt 1959). Thus, there is likely an abundant food supply for the heterotrophic dinoflagellates.

New Jersey Inland Bays (NJ), Delaware Bay (DE, NJ), and Delaware Inland Bays (DE) are unique due to the presence of *P. zoharyi*, a species not found at higher latitudes in the northwest Atlantic. *P. zoharyi* has an affinity for subtropical to tropical near‐shore environments (e.g., Usup et al. 2012). It only forms blooms in waters with temperatures >20°C and salinities >20 (Usup et al. 2012) and thus is restricted to the summer months in our study region where average summer temperatures are 22–23°C. New Jersey Inland Bays appears to be the northernmost limit for this species in the Western Atlantic, and the highest abundance (38–46%) is recorded in Delaware Inland Bays. *Pyrodinium bahamense*, the motile‐stage dinoflagellate responsible for producing *P. zoharyi* cysts, is toxic and can cause paralytic shellfish poisoning if in high enough concentrations. This species is known to occur in Delaware waters, but typically not in sufficient abundance to cause toxicological effects (Rouse 2007). Shallower water depths and longer residence times favor *P*. *bahamense* in Florida (Phlips et al. 2006) and may explain the greater abundance of this species in Delaware Inland Bays compared to Delaware Bay.

### The estuarine context in a changing climate

The estuarine context is important for understanding present‐day species distribution and the factors controlling them, in order to better predict how they may change in the future. The Gulf of Maine is experiencing effects of climate change, where melting Arctic sea ice is diluting salinity (Greene et al. 2008). Further salinity decreases are expected as climate models predict increased precipitation within watersheds of the Gulf of Maine (Wake et al. 2006). Adding freshwater affects stratification, nutrient dynamics, and ultimately phytoplankton production. Furthermore, in the southwest Atlantic, there is a trend toward more dinoflagellates and fewer diatoms, and there is also a shift in the timing of occurrence of these groups (Leterme et al. 2005). If SSTs in Delaware and New Jersey (Virginian Province) increase in the future, the northernmost limit of toxic *P. zoharyi* may extend to higher latitudes and bloom events in areas it already occupies may become more severe.

## Conclusions

This study encompasses 27 estuaries from an extensive geographic area, spanning 8° of latitude from Prince Edward Island (Canada) to Delaware (USA), and includes two biogeographic provinces. It is one of the first studies to directly assess the effect of different estuary types on dinoflagellate cyst assemblages. The cyst signal reflects the complex interplay between various water quality parameters. In northwest Atlantic estuaries, the main factors influencing the cyst assemblages are tidal range, SST, and SSS. Nutrients and sea ice cover were not measured in this study, but are known to influence dinoflagellates (e.g., de Vernal et al. 2005; Zonneveld et al. 2013).

The cyst signal clearly documents two biogeographic provinces and supports studies of other marine organisms which delineate the boundary of the Acadian and Virginian provinces at Cape Cod. Higher abundance of *Islandinium*? *cezare*,* Dubridinium* spp., and *Selenopemphix quanta* is found in the Acadian Province, while greater abundance of *Spiniferites* spp. and *Lingulodinium machaerophorum* is observed in the Virginian Province. Taxa such as *Polysphaeridium zoharyi*,* Echinidinium transparantum*, and *Trinovantedinium applanatum* have only been found in the Virginian Province. Providing a biogeographic context will better enable researchers and managers to interpret dinoflagellate cysts as indicators of human‐ and climatic‐induced stressors.

Estuary type has a major influence on the distribution of dinoflagellate cyst taxa within biogeographic provinces. Samples belonging to the same estuary type more closely resemble one another and cluster together in multivariate ordination. Some clusters are not as tight, in part due to intraestuary variability. Changes in precipitation patterns and temperature are likely to impact estuaries more directly than the open ocean due to their close proximity with land and shallower water depths.

## Conflict of Interest

None declared.

## Supporting information


**Appendix S1.** Supplementary methods.

**Appendix S2.** Supporting tables and figures.
**Table S1.** Taxonomic citation of dinoflagellate cysts identified in this study.
**Figure S1** Total cyst concentrations.
**Figure S2.** Maps showing the abundance of *Spiniferites* cf. *delicatus*,* Islandinium*? *cezare*,* Selenopemphix quanta*, and *Dubridinium* spp.
**Figure S3.** Maps showing the abundance of *Operculodinium centrocarpum*,* Brigantedinium* spp. and *Spiniferites* spp.
**Figure S4.** Maps showing the abundance of cysts of *Alexandrium* spp. and cysts of *Pentaphasodinium dalei*.
**Figure S5.** Maps showing the abundance of spiny brown cysts, SBC type P, SBC type E and *Islandinium minitum*.
**Figure S6.** Maps showing the abundance of cysts of *Polykrikos schwartzii*, cysts of *Polykrikos kofoidii*,* Lejeunecysta* spp., and *Spiniferites bentori*.
**Figure S7.** Maps showing the abundance of *Polysphaeridinium zoharyi*,* Quinquecuspsis concreta, Islandinium brevispinosum*, and *Achomasphaera* spp.
**Figure S8**. Maps showing the abundance of *Lingulodinium machaerophorum*,* Cyrodinium* sp., *Operculodinium israelianum*, and *Trinovantedinium applanatum*.
