# Supplementary material for: Biogeography of dinoflagellate cysts in northwest Atlantic estuaries
Source: Ecol Evol. 2016 Jul 19;6(16):5648–62. doi: 10.1002/ece3.2262 (PMC4983581; doi:10.1002/ece3.2262)
Supplement: Supplementary file 1 — Appendix S1. Supplementary methods. [file ECE3-6-5648-s001.docx]

**Appendix S1** Supplementary methods

*Brigantedinium* spp. includes those belonging to the *Brigantedinium* genus and round brown cysts where archeopyles were not observed. *Spiniferites* spp. includes all species of *Spiniferites* except *Spiniferites* *bentorii*, *Spiniferites elongatus*, *Spiniferites mirabilis*, *Spiniferites hypercanthus*, *Spiniferites membranaceus*, and *Spiniferites* cf. *delicatus*. *Selenopemphix quanta* and cysts of *Protoperidinium nudum* are grouped together as they are morphological very similar to each other. Cysts of *Polykrikos kofoidii* and *Polykrikos schwartzii* were identified following the nomenclature of Matsuoka et al. (2009). Spherical, dark brown reticulate cysts with a chasmic archeopyle, were classified as cysts of *Gymnodinium* *microreticulatum* due to their small body diameter (~22-27 μm). Round spiny brown cysts (SBCs) were identified according to Radi et al., 2013. Where they did not conform to previous descriptions, cyst types were assigned. SBC type PEI has a relatively large round (~36 μm diameter) central body with a cyst wall that is smooth to microreticulate. Processes are numerous, long (~ 8-13 μm), thin and hair-like, and are often bent. Process tips appear to be slightly expanded or capitate. SBC type P is a small (~ 20 μm) spiny brown cyst with a slight purplish hue, and is has a smooth and spherical central body. Processes are 5-10 μm in length, slender and are acuminate. SBC type E is a small spiny brown cyst with a spherical central body and appears to be the same cyst (cyst type E) depicted by Pospelova et al., (2002, their figure 6 g-h) and found in Massachusetts (USA). The central body is ~25 μm. Processes are 3-5 μm and many are in the center of paraplates. Cysts of cf. *Biecheleria* were enumerated but not included in the total cyst counts, due to their small size (e.g. Price and Pospelova, 2011).

Matsuoka, K., Kawami, H., Nagai, S., Iwataki, M. and Takayama, H. (2009). Re-examination of cyst–motile relationships of *Polykrikos kofoidii* Chatton and *Polykrikos schwartzii* Butschli (Gymnodiniales, Dinophyceae). *Review of Palaeobotany and Palynology, 154,* 79-90*.*

Price, A.M. and Pospelova, V. (2011). High-resolution sediment trap study of organic-walled dinoflagellate cyst production and biogenic silica flux in Saanich Inlet (BC, Canada). Marine Micropaleontology, 80(1-2),18-43.

Radi, T., Bonnet, S., Cormier, M.-A., de Vernal, A., Durantou, L., Faubert, É., Head, M.J., Henry, M., Pospelova, V., Rochon, A. and Van Nieuwenhove, N. (2013). Operational taxonomy and (paleo-)autecology of round, brown, spiny dinoflagellate cysts from the Quaternary of high northern latitudes. *Marine Micropaleontology, 98,* 41-57.
