# Supplementary material for: Biogeography of dinoflagellate cysts in northwest Atlantic estuaries
Source: Ecol Evol. 2016 Jul 19;6(16):5648–62. doi: 10.1002/ece3.2262 (PMC4983581; doi:10.1002/ece3.2262)
Supplement: Supplementary file 2 — Appendix S2. Supporting tables and figures. Table S1. Taxonomic citation of dinoflagellate cysts identified in this study. Figure S1 Total cyst concentrations. Figure S2. Maps showing the abundance of Spiniferites cf. delicatus, Islandinium? cezare, Selenopemphix quanta, and Dubridinium spp. Figure S3. Maps showing the abundance of Operculodinium centrocarpum, Brigantedinium spp. and Spiniferites spp. Figure S4. Maps showing the abundance of cysts of Alexandrium spp. and cysts of Pentaphasodinium dalei. Figure S5. Maps showing the abundance of spiny brown cysts, SBC type P, SBC type E and Islandinium minitum. Figure S6. Maps showing the abundance of cysts of Polykrikos schwartzii, cysts of Polykrikos kofoidii, Lejeunecysta spp., and Spiniferites bentori. Figure S7. Maps showing the abundance of Polysphaeridinium zoharyi, Quinquecuspsis concreta, Islandinium brevispinosum, and Achomasphaera spp. Figure S8. Maps showing the abundance of Lingulodinium machaerophorum, Cyrodinium sp., Operculodinium israelianum, and Trinovantedinium applanatum. [file ECE3-6-5648-s002.docx]

**Appendix S2** Supporting tables and figures

**Table S1.** Taxonomic citation of dinoflagellate cysts identified in this study.

| Cyst species  (paleontological name) | Dinoflagellate theca  (biological name) |
| --- | --- |
| **Autotrophic** |  |
| *Achomosphaera* spp*.* | ? *Gonyaulax* sp. Indet. |
| *-----* | *Alexandrium* spp. |
| *Ataxodinium choane* | *Gonyaulax spinifera* complex |
| *Bitectodinium spongium* | unknown |
| *Bitectodinium tepikiense* | *Gonyaulax spinifera* |
| *Impagidinium* spp. | *Gonyaulax* sp. Indet. |
| *Lingulodinium machaerophorum* | *Lingulodinium polyedra* |
| *Nematosphaeropsis labyrinthus* | *Gonyaulax spinifera* complex |
| *Operculodinium centrocarpum*  sensu Wall & Dale 1966 | *Protoceratium reticulatum* |
| *Operculodinium israelianium* | *?Protoceratium* sp*.* |
| ----- | *Pentapharsodinium dalei* |
| *Polysphaeridium zoharyi* | *Pyrodinium bahamense* |
| *Spiniferites elongatus* | *Gonyaulax elongata* |
| *Spiniferites membranaceus* | *Gonyaulax spinifera* complex |
| *Spiniferites belerius* | *Gonyaulax scrippsae, G. spinifera* complex |
| *Spiniferites ramosus* | *Gonyaulax scrippsae, G. spinifera* complex |
| *Spiniferites bentorii* | *Gonyaulax digitalis, G. spinifera* complex |
| *Spiniferites* cf. *delicatus* | *Gonyaulax spinifera* complex |
| *Spiniferites hyperacanthus* | *Gonyaulax spinifera* complex |
| *Spiniferites bulloideus* | *Gonyaulax scrippsae, G. spinifera* complex |
| *Spiniferites mirabilis* | *Gonyaulax spinifera* complex |
| *Spiniferites* spp. | *Gonyaulax* complex |
| *Spiniferites* type 1. | *Gonyaulax* complex |
| *Tectadodinium pellitum* | *Gonyaulax spinifera* complex |
| *Tuberculodinium vancampoae* | *Pyrophacus steinii* |
| **Heterotrophic** |  |
| *Dubridinium* spp. | Diplopsalid group |
| ----- | *Gymnodinium* spp*.* |
| ----- | *Gymnodinium micorecitulatum* |
| ----- | *Polykrikos kofoidii* |
| ----- | *Polykrikos schwartzii* |
| ----- | *Protoperidinium oblongum* |
| ----- | *Protoperidinium paraoblongum* |
| ----- | *Protoperidinium quadrioblongum* |
| *Brigantedinium* spp. | *?Protoperidinium* spp. |
| *Brigantedinium cariacoense* | *Protoperidinium avellana* |
| *Brigantedinium simplex* | *Protoperidinium conicoides* |
| *Cyrodinium sp.* | unknown |
| *Echinidinium delicatum* | *Protoperidinium* sp. indet. |
| *Echinidinium transparantum* | *Protoperidinium* sp. indet. |
| *Echinidinium* cf. *zonneveldii* | *Protoperidinium* sp. indet. |
| *Echinidinium* spp. | *Protoperidinium* sp. indet. |
| *Islandinium minutum* | *Protoperidinium* sp. indet. |
| *Islandinium? cezare* | *Protoperidinium* sp. indet. |
| *Islandinium brevispinosum* | *Protoperidinium* sp. indet. |
| *Lejeunecysta marina* | *Protoperidinium* sp. indet. |
| *Lejeunecysta oliva* | *Protoperidinium* sp. indet. |
| *Lejeunecysta sabrina* | *Protoperidinium* sp. indet. |
| *Lejeunecysta* spp*.* | *Protoperidinium* sp. indet. |
| *-----* | *Peridinium minutum* sensu Wall and Dale 1968 |
| ----- | *Protoperidinium americanum* |
| ----- | *Protoperidinium nudum* |
| ----- sensu Wall and Dale 1968 | *Protoperidinium oblongum* |
| *Protoperidinium* spp. | *Protoperidinium* spp. indet. |
| *Quinquecuspis concreta* | *Protoperidinium leonis* |
| *Selenopemphix nephroides* | *Protoperidinium* sp. indet. |
| *Selenopemphix quanta* | *Protoperidinium conicum* |
| *Stelladinium reidii* | *Protoperidinium compressum* |
| *Trinovantedinium applanatum* | *Protoperidinium pentagonum* |
| *Votadinium calvum* | *Protoperidinium oblongum* |
| *Votadinium spinosum* | *Protoperidinium claudicans* |
| Cyst type PEI | unknown |
| Cyst type E | unknown |
| Cyst type P | unknown |
| Spiny brown cysts | *?Protoperidinium* sp. indet. |
|  |  |

**Figure S1** Total cyst concentrations.


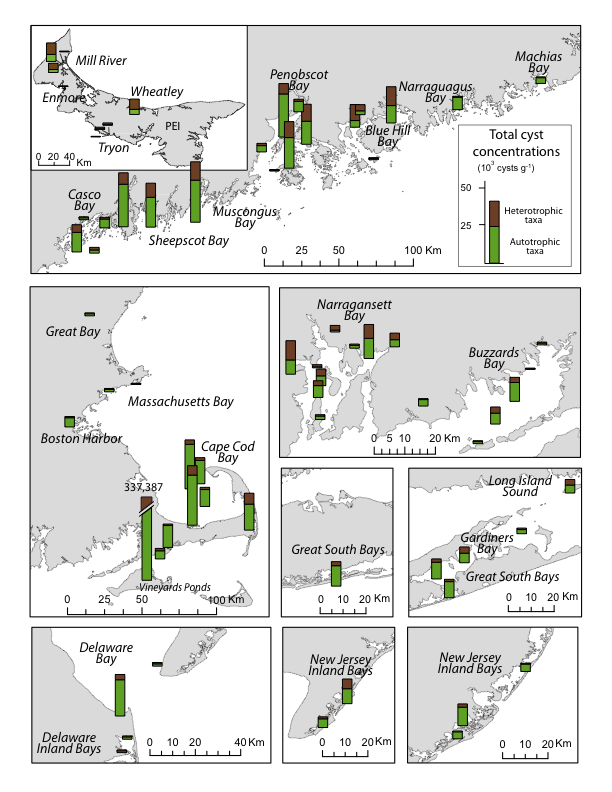


**
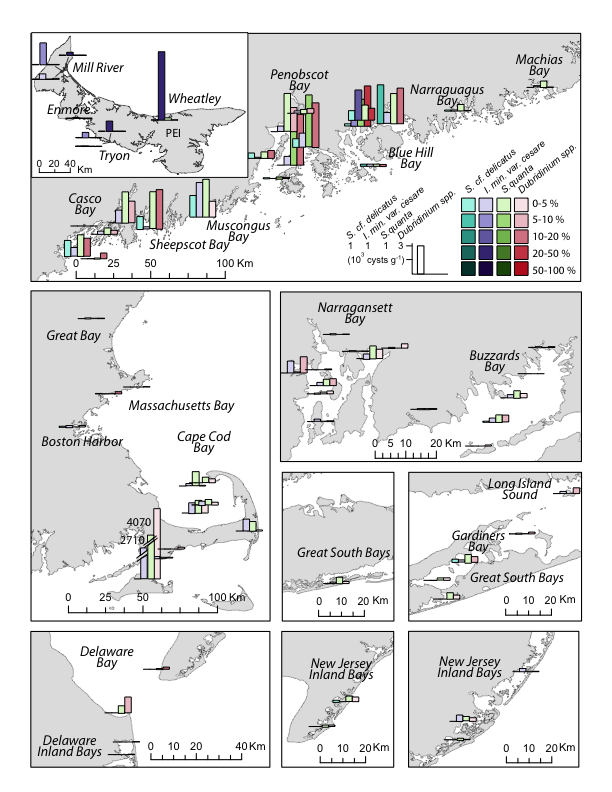
Figure S2.** Maps showing the abundance of *Spiniferites* cf. *delicatus*, *Islandinium*? *cezare*, *Selenopemphix quanta*, and *Dubridinium* spp. The height of each bar represents cyst concentrations (cysts g^-1^) and the scale differs between species. Different shades represent differences in cyst relative abundances (%), where darker shades indicate higher abundances.

**
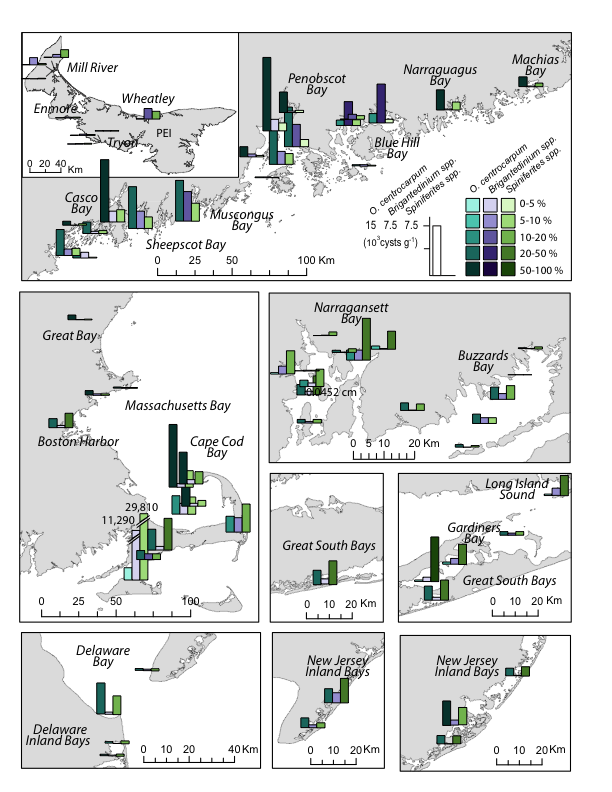
Figure S3.** Maps showing the abundance of *Operculodinium centrocarpum*, *Brigantedinium* spp. and *Spiniferites* spp. The height of each bar represents cyst concentrations (cysts g^-1^) and the scale differs between species. Different shades represent differences in cyst relative abundances (%), where darker shades indicate higher abundances.

**
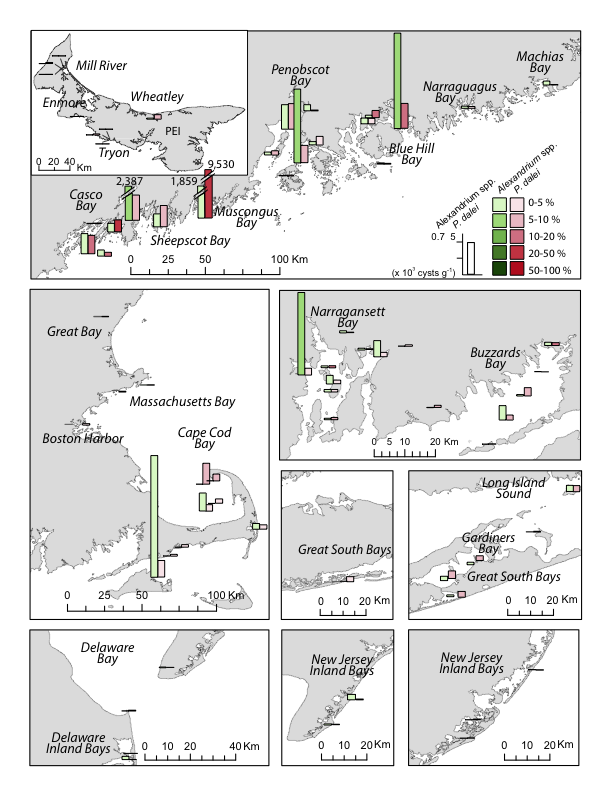
Figure S4.** Maps showing the abundance of cysts of *Alexandrium* spp. and cysts of *Pentaphasodinium dalei*. The height of each bar represents cyst concentrations (cysts g^-1^) and the scale differs between species. Different shades represent differences in cyst relative abundances (%), where darker shades indicate higher abundances.

**
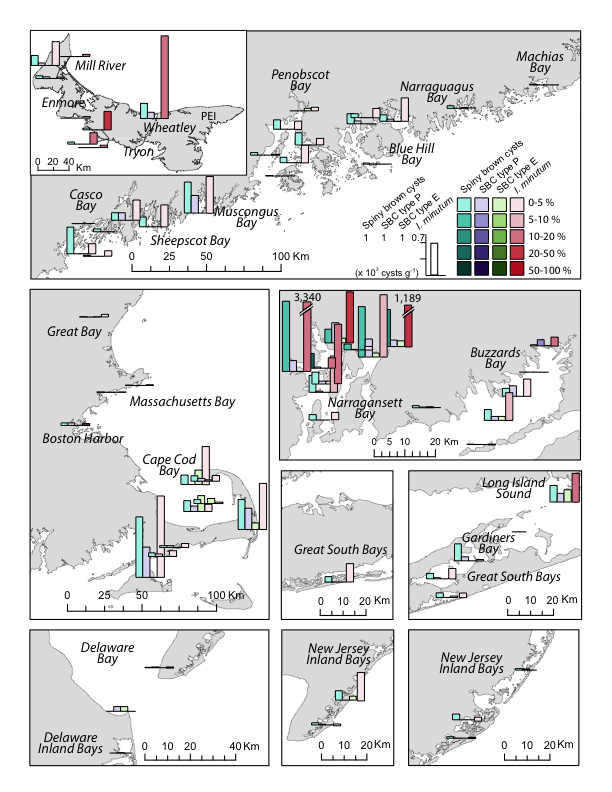
Figure S5.** Maps showing the abundance of spiny brown cysts, SBC type P, SBC type E and *Islandinium minitum*. The height of each bar represents cyst concentrations (cysts g^-1^) and the scale differs between species. Different shades represent differences in cyst relative abundances (%), where darker shades indicate higher abundances.

**
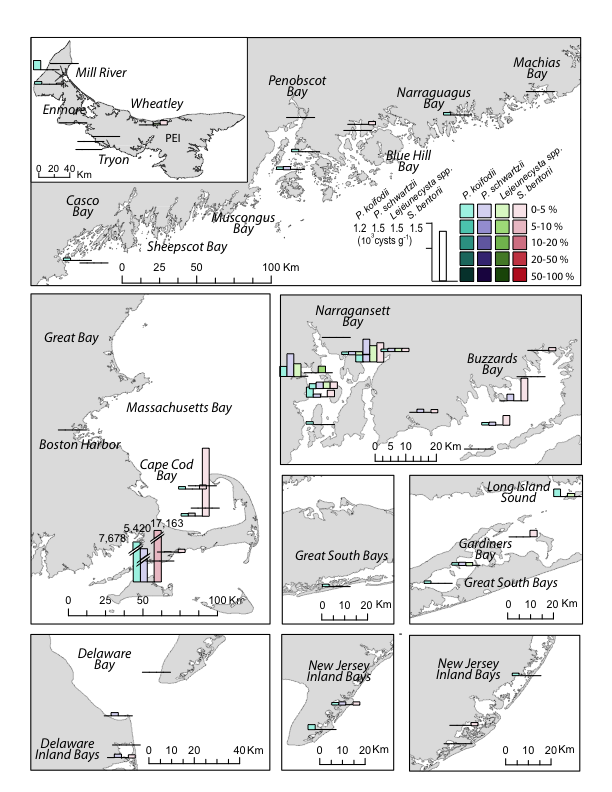
Figure S6.** Maps showing the abundance of cysts of *Polykrikos schwartzii*, cysts of *Polykrikos kofoidii*, *Lejeunecysta* spp., and *Spiniferites bentori*. The height of each bar represents cyst concentrations (cysts g^-1^) and the scale differs between species. Different shades represent differences in cyst relative abundances (%), where darker shades indicate higher abundances.

**
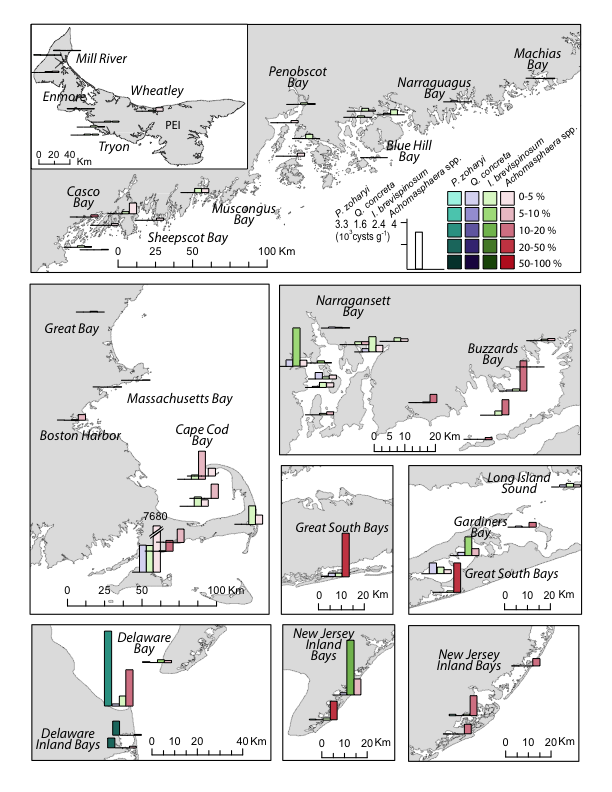
Figure S7.** Maps showing the abundance of *Polysphaeridinium zoharyi*, *Quinquecuspsis concreta,* *Islandinium brevispinosum*, and *Achomasphaera* spp. The height of each bar represents cyst concentrations (cysts g^-1^) and the scale differs between species. Different shades represent differences in cyst relative abundances (%), where darker shades indicate higher abundances.

**
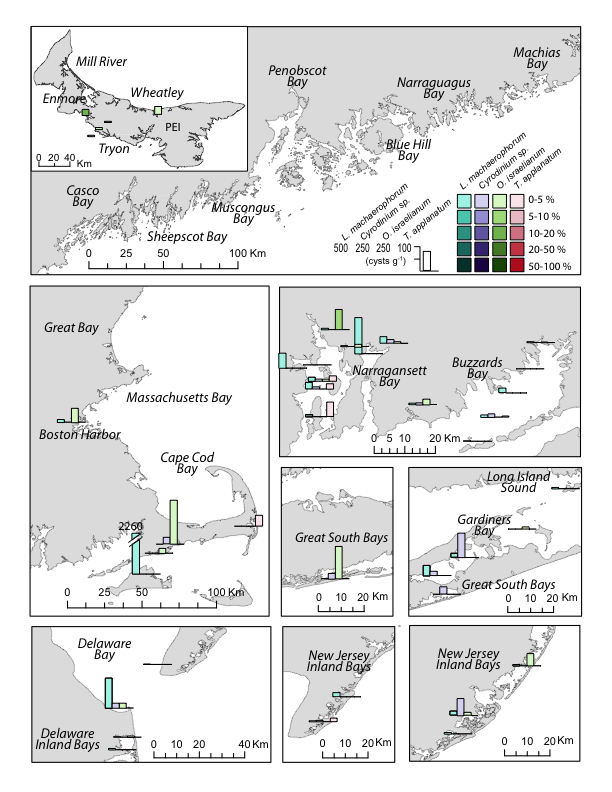
Figure S8**. Maps showing the abundance of *Lingulodinium machaerophorum*, *Cyrodinium* sp., *Operculodinium israelianum*, and *Trinovantedinium applanatum*. The height of each bar represents cyst concentrations (cysts g^-1^) and the scale differs between species. Different shades represent differences in cyst relative abundances (%), where darker shades indicate higher abundances.
